# Macrophage Control of Incipient Bone Formation in Diabetic Mice

**DOI:** 10.3389/fcell.2020.596622

**Published:** 2021-01-25

**Authors:** Miya Kang, Ghadeer Thalji, Chun-Chieh Huang, Sajjad Shirazi, Yu Lu, Sriram Ravindran, Lyndon F. Cooper

**Affiliations:** ^1^Department of Oral Biology, College of Dentistry, University of Illinois at Chicago, Chicago, IL, United States; ^2^Department of Restorative Dentistry, College of Dentistry, University of Illinois at Chicago, Chicago, IL, United States

**Keywords:** osteogenesis, db/db mouse, macrophage, bone regeneartion, type 2 diabetes mellitus

## Abstract

Both soft and hard tissue wound healing are impaired in diabetes. Diabetes negatively impacts fracture healing, bone regeneration and osseointegration of endosseous implants. The complex physiological changes associated with diabetes often manifest in immunological responses to wounding and repair where macrophages play a prominent role in determining outcomes. We hypothesized that macrophages in diabetes contribute toward impaired osseous wound healing. To test this hypothesis, we compared osseous wound healing in the mouse calvaria defect model using macrophages from C57BL/6J and db/db mice to direct osseous repair in both mouse strains. Initial analyses revealed that db/db mice macrophages showed an inflamed phenotype in its resting state. Incipient bone regeneration evaluated by μCT indicated that bone regeneration was relatively impaired in the db/db mouse calvaria and in the calvaria of C57BL/6J mice supplemented with db/db macrophages. Furthermore, osteogenic differentiation of mouse mesenchymal stem cells was negatively impacted by conditioned medium from db/db mice compared to C57BL/6J mice. Moreover, miR-Seq analysis revealed an altered miRNA composition in db/db macrophages with up regulated pro-inflammatory miRNAs and down regulated anti-inflammatory miRNAs. Overall, this study represents a direct step toward understanding macrophage-mediated regulation of osseous bone regeneration and its impairment in type 2 diabetes mellitus.

## Introduction

Type 2 diabetes mellitus (T2DM) is a chronic disease of growing prevalence worldwide that has broad and severe systemic consequences (Geiss et al., 2014; IDF Diabetes Atlas, 2020). The impact of altered insulin secretion and insulin resistance on the health of individuals has far-reaching implications that are frequently highlighted by cutaneous ulcers, heart disease, and kidney failure. Elevated inflammation is associated with and, in part, contributory to this pathology (Daryabor et al., 2020). T2DM also influences bone physiology. This is evidenced with respect to bone remodeling and increased risk of bone fractures (Fan et al., 2016), by altered bone density, reduced bone turnover (Reyes-García et al., 2013), and reduced bone repair and regeneration (Follak et al., 2005; Hu et al., 2018). While studies indicate that T2DM influences osteoclastogenesis (Kasahara et al., 2010), hyperglycemia associated with T2DM can influence osteoblastic differentiation (Phimphilai et al., 2017) to affect bone healing (Bhamb et al., 2019). In a human clinical study using T2DM and body mass index (BMI)-matched control subjects, peripheral blood osteoblastic precursors were significantly reduced in T2DM subjects (*P* = 0.0007) (Sassi et al., 2018). Human osteoblastic cell cultures obtained from hip fracture patients demonstrated that T2DM patients showed marked reduction in *RUNX2* and *OSTERIX* gene expression when exposed to high glucose (Miranda et al., 2016). The impact of diabetes on osteoblast function and the related altered inflammatory signaling remains a central focus of attention.

Several possible mechanisms have been explored concerning how T2DM influences bone physiology, quality, and function. Included are the roles of insulin resistance, enhanced marrow adiposity, formation of advanced glycation end products (AGEs), increased reactive oxygen species, and altered inflammatory factors (Jiao et al., 2015; Chiodini et al., 2020). In T2DM associated with obesity, there is chronic low-grade inflammation and accumulation of pro-inflammatory cells in visceral fat and inflammatory stimulation of tissue macrophages (Wensveen et al., 2015). In fact, the secretion of inflammatory cytokines by multiple cell types is strongly implicated in the inflammatory pathogenesis associated with T2DM (Nikolajczyk et al., 2011). Macrophages are central regulators of inflammation and secrete many of the proinflammatory cytokines and chemokines that direct the inflammatory effects observed in T2DM. They are further required for regulation of wound healing that is well-known to be diminished in T2DM. In cutaneous wounds of diabetic mice, there is increased accumulation of macrophages and sustained accumulation of inflammatory macrophages. The macrophages of diabetic mice are M1-like macrophages, further suggesting an elevated inflammatory phenotype that may impair the regenerative phase of wound healing (Barman and Koh, 2020). The T2DM-associated alteration in macrophage phenotype may also play a role in the regulation of bone repair and regeneration.

Several studies implicate macrophages and their polarization in the regulation of bone regeneration. This may result from the induction of an M2 or wound healing macrophage phenotype (Shi et al., 2018; Wang et al., 2018; Bai et al., 2020). When M2 macrophage-derived extracellular vesicles (EVs) were added to rat calvarial defects within a collagen scaffold, bone healing was increased at 3 and 6 weeks compared to naïve macrophages or M1 EVs (Kang et al., 2020). Given the impact of T2DM on macrophage phenotype and the role that macrophages play in modulating osteogenesis, it is possible that macrophages contribute to the pathophysiology of impaired bone regeneration in T2DM. While several mouse models are available for T2DM-related research including chemical induction of T2DM (King, 2012), the db/db mouse model has been studied with respect to post-natal bone regeneration and serves as a good model for T2DM. Both ectopic osteogenesis and fracture repair have been studied. Delayed mesenchymal osteogenesis and impaired microvascularization were observed (Roszer et al., 2014). In this report, the effect of macrophages on calvarial bone repair was examined in the db/db mouse to investigate the effects of T2DM on bone regeneration.

## Materials and Methods

### Isolation of Bone Marrow-Derived Macrophages

Bone marrow-derived macrophages were isolated from 6-week-old C57BL/6J wild-type (WT) mice (The Jackson Laboratory) and BKS.Cg-Dock7^m^+/+Lepr^db^/J db/db (DB) mice (The Jackson Laboratory) as described in Mirza et al. (2013). Briefly, bone marrow cells were flushed out of the mouse femur and tibia and passed through a 40-μm cell strainer. The cells were seeded in 100-mm culture dishes and cultured in Dulbecco's Modified Eagle Medium (DMEM, Gibco) containing 20% fetal bovine serum (FBS, Gibco) and 1% antibiotic–antimycotic solution (Gibco). WT macrophages and DB macrophages were obtained by adding 20 ng/ml of recombinant murine M-CSF (PeproTech) into the DMEM growth medium for 7 days. The macrophages were detached by gently pipetting ice-cold phosphate-buffered saline (PBS) containing 5% FBS across the dish followed by incubation at 4°C for 10 min. The cells were then centrifuged at 300 × *g* for 5 min and resuspended in DMEM growth medium for further experiments. For inflammatory stimuli to be induced, macrophages were treated with 100 ng/ml of lipopolysaccharide (LPS) for 24 h.

### Flow Cytometry

WT and DB macrophages were preincubated with rat monoclonal [93] TruStain FcX™ anti-CD16/CD32 antibody (101320, BioLegend) to block the Fc receptor prior to further staining. Cell surface antigens were labeled with rat monoclonal [D7] PE/Cyanine7 anti-mouse Ly-6A/E (Sca-1) antibody (108114, BioLegend), rat monoclonal [1A8] Alexa Fluor® 647 anti-mouse Ly-6 antibody (127610, BioLegend), and rat monoclonal [BM8] PE anti-mouse F4/80 antibody (123110, BioLegend). Samples were subjected to analysis using a Gallios flow cytometer and Kaluza software.

### Immunocytochemistry

WT and DB macrophages were seeded in 12-well culture dishes and incubated for 18 h at 37°C in 5% CO_2_ for immunocytochemistry staining. The coverslips were fixed in 4% paraformaldehyde (PFA) for 15 min, permeabilized using 0.1% Triton X-100 (Fisher Scientific) for 10 min, and blocked with 5% bovine serum albumin (BSA) for 1 h at room temperature. Following incubation with rat monoclonal [CI:A3-1] anti-F4/80 antibody (1/100, ab6640, Abcam), rabbit monoclonal anti-inducible nitric oxide synthase (iNOS) antibody (1/100, ab15323, Abcam), rabbit monoclonal anti-mannose receptor (CD206) antibody (1/100, ab64693, Abcam), mouse monoclonal [3A6] anti-interleukin-1 beta (IL-1β) antibody (1/100, 12242, Cell Signaling), and mouse monoclonal [AC-15] anti-beta actin antibody (1/1,000, NB600-501, Novus Biologicals) overnight at 4°C, cells were then treated with fluorescein isothiocyanate (FITC)- and tetramethylrhodamine (TRITC)-conjugated secondary antibodies (1/1,000, Sigma) for 1 h at room temperature. Cells were imaged using a Zeiss LSM 710 Meta confocal microscope. For quantification of iNOS and IL-1β staining, ImageJ software was used to calculate the fluorescence intensity. The values were presented as normalized fluorescence intensity divided by the number of nuclei per field (*n* = 4 per group).

### qRT PCR

Total RNA was isolated using an RNeasy Mini Kit (Qiagen) as per the manufacturer's protocol. The RNA concentration was measured using NanoDrop One. After first-strand cDNA synthesis was completed, gene-specific primers (Table 1) were used to direct PCR amplification and SYBR Green probe incorporation using a Bio-Rad CFX96 thermocycler. All expression data were normalized to housekeeping genes GAPDH, and fold change was calculated using the ^ΔΔ^Ct method (*n* = 4 per group).

**Table 1:** Primer pairs used for qRT PCR.

**Genes**	**Forward (5^**′**^-3^**′**^)**	**Reverse (5^**′**^-3^**′**^)**	**Size**
			**(bp)**
GAPDH	AGGTCGGTGTGAACGGATTTG	GGGGTCGTTGATGGCAACA	123
IL-1β	GCAACTGTTCCTGAACTCAACT	ATCTTTTGGGGTCCGTCAACT	89
IL-6	TAGTCCTTCCTACCCCAATTTCC	TTGGTCCTTAGCCACTCCTTC	76
TNFα	CAGGCGGTGCCTATGTCTC	CGATCACCCCGAAGTTCAGTAG	89
IL-10	GCTCTTACTGACTGGCATGAG	CGCAGCTCTAGGAGCATGTG	105
BMP2	GGGACCCGCTGTCTTCTAGT	TCAACTCAAATTCGCTGAGGAC	154
RUNX2	ATGCTTCATTCGCCTCACAAA	GCACTCACTGACTCGGTTGG	146
OSX	ATGGCGTCCTCTCTGCTTG	TGAAAGGTCAGCGTATGGCTT	156

### Enzyme-Linked Immunosorbent Assay (ELISA)

Cell culture supernatants were collected from WT and DB macrophages and centrifuged at 1,500 rpm for 10 min. The supernatants were then subjected to an ELISA for detection of cytokine secretion. Mouse IL-6 ELISA kit (Invitrogen) and mouse tumor necrosis factor alpha (TNFα) ELISA kit (Invitrogen) were used per the manufacturer's protocol. The protein concentration of the culture supernatant was measured using a BCA Protein Assay Kit (Thermo Fisher Scientific), and an equal amount of protein was added to each well. The absorbance was measured at 450 nm, and values were presented as concentration in pg/ml according to the standard curve (*n* = 3 per group).

### Phagocytosis Assay

Phagocytic activity of WT and DB macrophages were compared using a Vybrant™ Phagocytosis Assay Kit (Invitrogen) as per the manufacturer's recommended protocol. Briefly, WT and DB macrophages were seeded onto 96-well culture dishes (1 × 10^5^ cells per well, *n* = 5 per group). The cells were incubated with fluorescently labeled *Escherichia coli* particles for 2 h and stained with trypan blue. The phagocytosis activity was quantitated by following the internalization of the fluorescent bioparticles, and the relative fluorescence units (RFUs) were measured at 480 nm using a fluorescence plate reader (BioTek plate reader).

### Mouse Calvarial Bone Defect Model

Mid-skull transcortical defects were created in 8-week-old mice using a 3.5-mm trephine dental drill. All the defects were filled with collagen scaffolds (BioPlug, BioHorizons). In the experimental groups, scaffolds were populated with 1 × 10^6^ of either WT macrophages (DB+wtMO group) or DB macrophages (WT+dbMO group) per defect. An equivalent volume of PBS was added in the scaffolds of WT and DB groups as a sham-treated control.

After 4 weeks, the mice were sacrificed by carbon dioxide asphyxiation followed by cervical dislocation. The calvariae were harvested, fixed in neutral buffered 4% PFA, and subjected to 3D μCT analysis using a Scanco40 μCT scanner. The μCT scanner data were analyzed using a custom-built Matlab program. All procedures were performed according to animal protocols approved by the Animal Care Committee of the Office of Animal Care and Institutional Biosafety (OACIB) of the University of Illinois at Chicago.

### Histology and Immunohistochemistry (IHC)

The calvariae were decalcified in 10% EDTA solution, embedded in paraffin, and sectioned into 5- to 10-μm sections. Hematoxylin and eosin (H&E) staining was performed as per previously published protocols (Huang et al., 2020).

For immunofluorescent staining, the slides were pre-treated with 5% BSA blocking buffer for 1h at room temperature and stained for osteomarkers and macrophage-specific antigens using mouse monoclonal [65529.111] anti-bone morphogenetic protein 2 (BMP2) antibody (1/100, ab6285, Abcam), mouse monoclonal [OCG3] anti-osteocalcin (OCN) antibody(1/100, ab13420, Abcam), rabbit monoclonal anti-iNOS antibody (1/100, ab15323, Abcam), rabbit monoclonal anti-CD206 antibody (1/100, ab64693, Abcam), and mouse monoclonal [3A6] anti-IL-1β antibody (1/100, 12242, Cell Signaling). Sections were then stained with anti-mouse FITC and anti-rabbit TRITC secondary antibodies (1/200, Sigma), imaged using Zeiss LSM 710 laser scanning confocal microscope equipped with Zen image analysis software. For a vascular marker, sections were stained with rabbit polyclonal anti-CD31 antibody (1/100, ab28364, Abcam) and peroxidase-conjugated secondary antibody. ImageJ was used to measure positive immunostained cell number or % area per field (*n* = 4 per group). The positive cell number of iNOS and CD206 was divided by the number of nuclei per field.

### Alkaline Phosphatase (ALP) Assay

Human bone marrow-derived mesenchymal stem cells (hMSCs) were purchased from Lonza. hMSCs (5 × 10^4^ cells per well) were seeded in 12-well tissue culture plates and cultured in αMEM (Gibco) containing 20% FBS, 1% antibiotic–antimycotic solution, and 1% l-glutamine (Gibco). Osteogenic differentiation was induced by culturing the cells in αMEM growth medium containing 100 μg/ml of ascorbic acid (Sigma), 10 mM β-glycerophosphate (Sigma), and 10 mM dexamethasone (Sigma) for 5 days.

For preparation of conditioned medium, WT and DB macrophages were seeded into T-25 tissue culture flasks (3 × 10^6^ cells per flask) in DMEM growth medium containing 20 ng/ml of M-SCF overnight. The cells were washed in growth medium and cultured under DMEM−1% FBS condition for 48 h. The culture medium was then harvested and centrifuged at 3,000 × g for 15 min to remove cell debris and added to the hMSCs at an osteogenic medium (OS)-to-CM ratio of 1:1.

hMSCs cultured in osteogenic medium were collected from each well at day 1 and 5. ALP activity was quantified using Alkaline Phosphatase Assay Kit (Abcam) by measuring *p*-nitrophenyl (*p*NP) based on the spectrophotometric absorbance at 405 nm. The fold change of ALP activity at day 5 was calculated with respect to the relative enzymatic activity of day 1.

### miRNA Sequencing (miR-Seq) Analysis and Quantitative miRNA Expression in Macrophages

RNA isolation from WT and DB macrophages was performed using a miRNeasy Mini Kit (Qiagen) as per the manufacturer's protocol. miR-Seq libraries were constructed using a QIAseq miRNA Library Kit (Qiagen) and sequenced on a NovaSeq 6000 at the UIC Core Genomics Facility. Fastq files were generated with the bclfastq v1.88.4, and adapter sequences and low-quality sequences were removed. miRNAs were identified with BWA ALN.

For qRT PCR, the exact same amounts of miRNA from WT and DB macrophages were utilized to complete cDNA synthesis with a miScript II RT Kit (Qiagen). qRT PCR was performed using a miScript SYBR Green PCR Kit (Qiagen) with custom primer for miR-155-5p: 5′-GGGTTAATGCTAATTGTGATAGGGGT-3′. Relative miRNA expression levels were normalized to RNU6B, and fold change was calculated using the ^ΔΔ^Ct method (*n* = 4 per group).

### Statistical Analysis

For experiments involving two groups, Student's *t*-test with a confidence interval of 95% was utilized. For the experiments involving comparison of more than two groups, one-way ANOVA was performed with a confidence interval of 95%, followed by pairwise comparisons using Tukey's *ad hoc* method (*P* < 0.05).

## Results

### Resting State of WT and DB Macrophages

**Figure 1:**
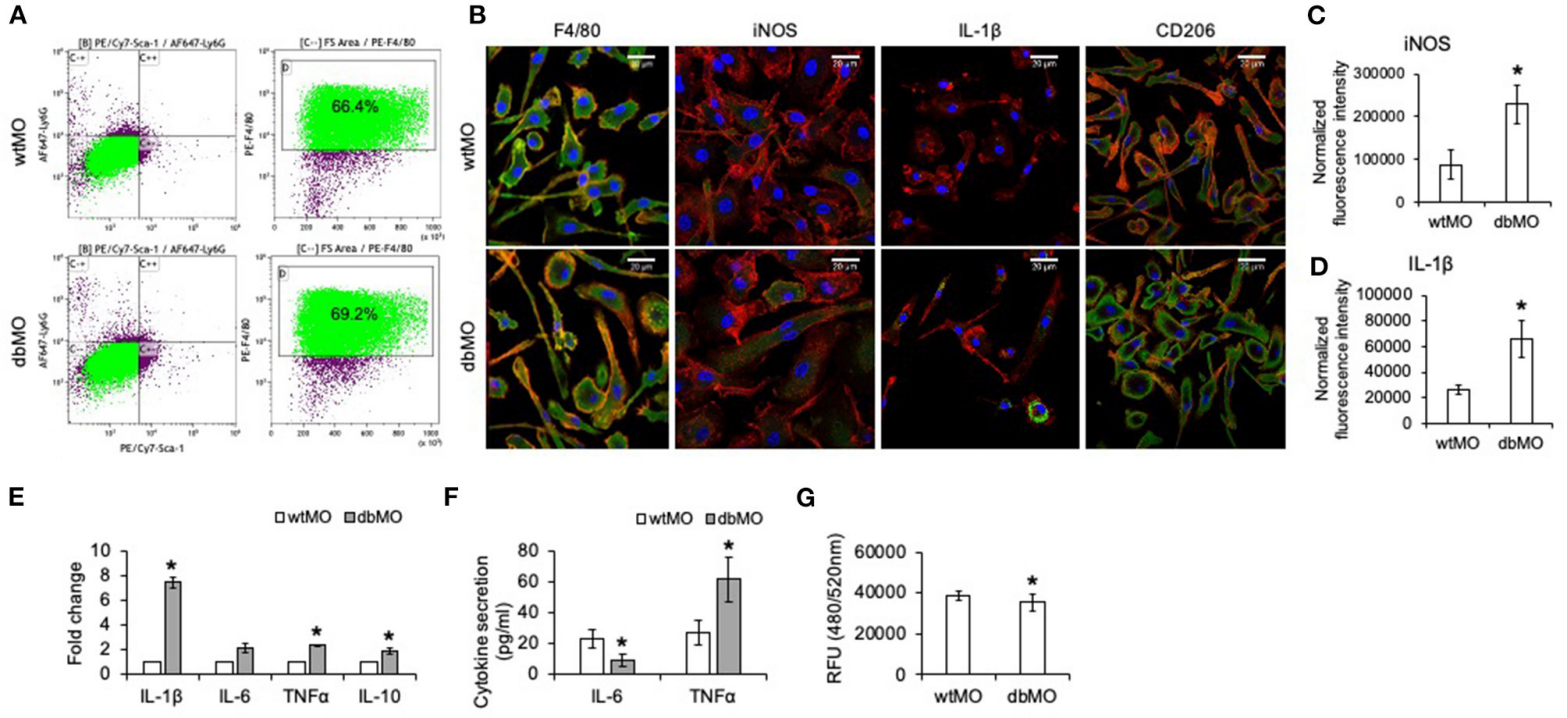
Characterization of WT and DB macrophages. **(A)** Flow cytometric analysis of Sca-1^−^/Ly6G^−^/F4/80^+^ cell population in macrophages isolated from WT and DB mice (wtMO and dbMO, respectively). **(B)** Immunocytochemistry of WT and DB macrophages for F4/80 (green), iNOS (green), CD206 (green), and IL-1β (green). Scale bar represents 20 μm in all images. **(C,D)** Normalized fluorescence intensity of iNOS and IL-1β in immunocytochemistry images using ImageJ software. **(E)** Quantitative RT PCR of WT and DB macrophages for inflammatory cytokines (IL-1β, IL-6, TNFα, and IL-10). The data represent fold change in DB macrophages with respect to WT macrophages (*n* = 4). Note the significantly increased expression of IL-1β in DB macrophages. **(F)** Quantification of IL-6 and TNFα in supernatants from WT and DB macrophages using ELISA (*n* = 3). **(G)** Phagocytosis assay with WT and DB macrophages (*n* = 5). The data represent RFUs. *Statistical significance (*P* < 0.05) calculated by Student's *t*-test.

We first demonstrated that macrophages isolated from WT and DB mice were similar. Both WT and DB macrophages demonstrate similar surface antigen profiles representative of macrophages as assessed by flow cytometry at the level of Sca-1, Ly6G, and F4/80 (Figure 1A). We next analyzed the resting states of WT and DB macrophages at the level of prominent inflammatory mediator expression.

Immunocytochemistry qualitatively affirmed the expression of iNOS and IL-1β only in DB macrophages and of CD206 in both WT and DB macrophages (Figure 1B). Furthermore, quantitation of protein expression from immunocytochemical staining in WT and DB macrophages indicated elevated expression of iNOS and IL-1β in the DB macrophages (Figures 1C,D). qRT PCR analysis quantitatively demonstrated the differences in the resting inflammatory states of the isolated WT and DB macrophages. Quantitative gene expression analysis of inflammatory cytokines showed that, when compared to WT macrophages, the DB macrophages possess significantly elevated proinflammatory cytokines including a higher expression of IL-1β (Figure 1E). ELISA for the other inflammatory cytokines IL-6 and TNFα indicated the reduced expression of IL-6 and an elevated presence of TNFα in the DB macrophages (Figure 1F). To observe if the phagocytic ability of the macrophages is altered, a phagocytosis assay was performed. Results presented in Figure 1G indicate a small albeit statistically significant reduction in the phagocytic activity of the DB macrophages.

**Figure 2:**
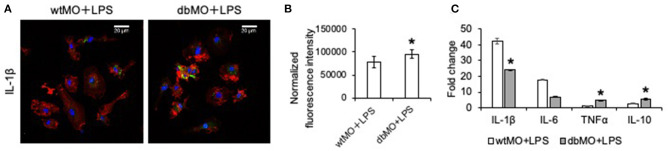
Response of WT and DB macrophages to inflammatory cytokine. **(A)** Representative confocal images of LPS-treated WT and DB macrophages (wtMO and dbMO, respectively), immunostained for IL-1β (green). Scale bar represents 20 μm in all images. **(B)** Normalized fluorescence intensity of IL-1β in immunocytochemistry images (*n* = 4). **(C)** Quantitative RT PCR of WT and DB macrophages for IL-1β, IL-6, TNFα, and IL-10. The data represent fold change in LPS-treated WT and DB macrophages with respect to untreated WT macrophages (*n* = 4). *Statistical significance (*P* < 0.05) calculated by Student's *t*-test.

To evaluate if the behavior of WT and DB macrophages is altered when stimulated for an inflammatory response, we treated both types of cells with *E. coli* LPS. Results presented in Figure 2 demonstrate that DB macrophages show increased presence of IL-1β when subjected to LPS stimulation (Figures 2A,B). They also show elevated gene expression levels of TNFα and IL-10 (Figure 2C). However, the gene expression levels of IL-1β were less than that of WT macrophage, indicating that the elevated protein expression could be a result of a translationally controlled phenomenon.

### Diabetic Macrophages Affect the Quality of Bone Regeneration

**Figure 3:**
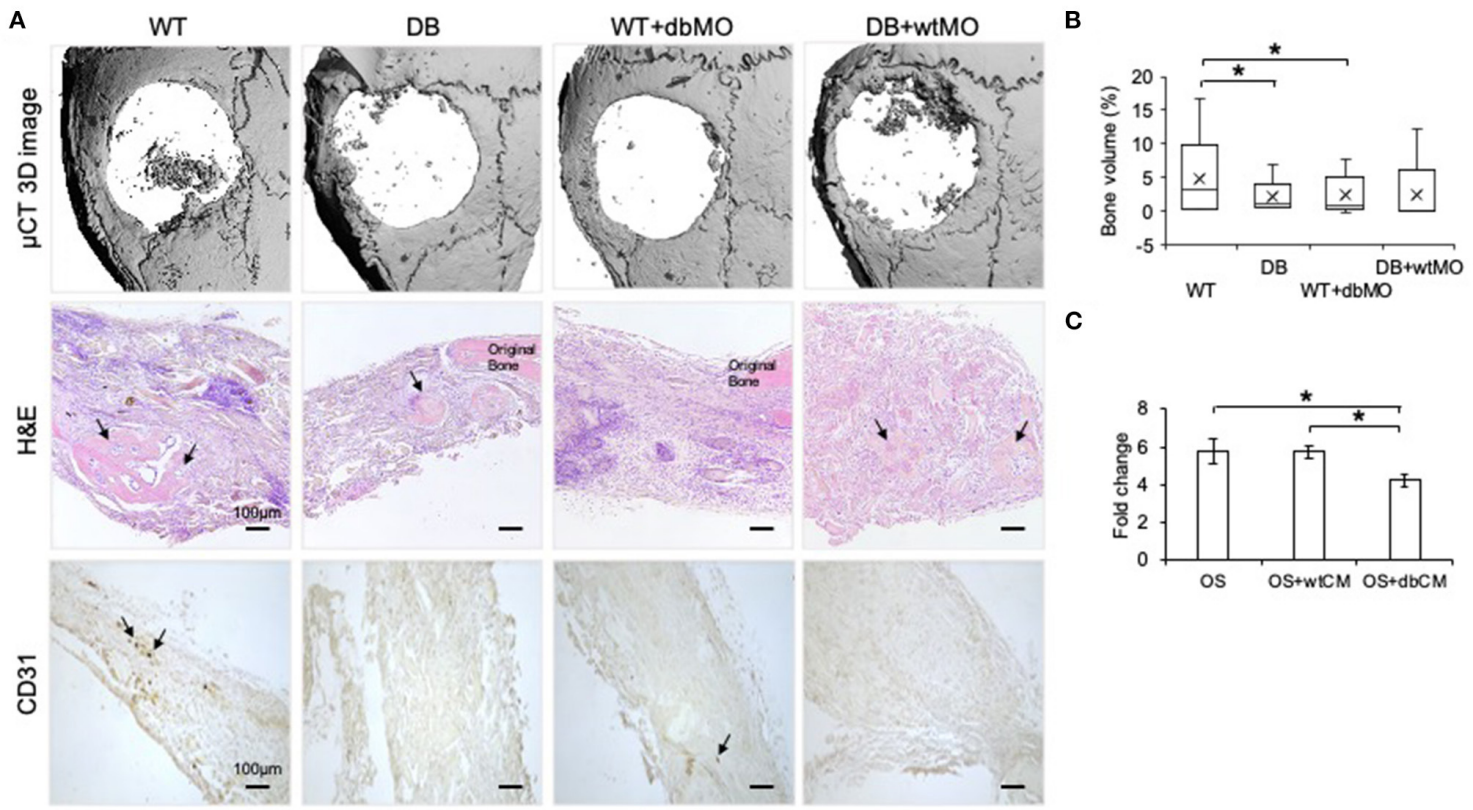
Diabetic macrophages affect the quality of bone regeneration. **(A)** Representative 3D μCT images and light microscopic images of the demineralized and paraffin-embedded tissue sections of the mouse calvaria at 4 weeks post wounding stained with hematoxylin and eosin (H&E) and 3,3′-diaminobenzidine (DAB) for CD31. WT, wild-type mice; DB, db/db mice; WT+dbMO, WT mice treated with DB macrophages; DB+wtMO, DB mice treated with WT macrophages. The black arrows represent newly formed bone in the H&E images and CD31-positive cells in DAB staining images. Scale bar represents 100 μm in all images. **(B)** Volumetric quantitation of the 3D μCT data (*n* = 6). The graph represents mean percentage bone volume regenerated to total volume of the defect ± SD. **(C)** The fold change represents alkaline phosphatase (ALP) activity in hMSCs treated with osteogenic medium (OS), osteogenic medium with WT macrophage conditioned medium (OS+wtCM), and osteogenic medium with conditioned medium from DB macrophages (OS+dbCM) with respect to hMSCs in growth medium at day 5 (*n* = 3). *Statistical significance (*P* < 0.05) as measured by Tukey's *ad hoc* test post ANOVA.

Macrophages are capable of modulating osteogenesis (Champagne et al., 2002; Sinder et al., 2015). We proceeded to investigate if incipient bone formation was affected by the DB macrophage phenotype. In the mouse calvarial defect model, we observed by quantitative μCT measurements that the incipient healing at 4 weeks significantly reduced in DB mice compared to WT mice (Figures 3A,B). When DB macrophages were introduced into the WT wound sites adherent to collagen scaffolds, they negatively impacted the healing of the WT mouse defects. Conversely, the introduction of WT macrophages into diabetic wounds improved healing. Together with the accompanying histological representation of absence of incipient bone formed in both the DB mouse and WT mouse treated with DB macrophages (Figure 3A), these results indicate that DB macrophages impair incipient bone formation. This impairment of osteogenesis in the DB mouse calvaria was partially reversed by the treatment of DB mouse calvarial defects with WT macrophages, further implying a role for the macrophages in the regulation of bone repair.

When the sections were probed with CD31 antibody for the presence of endothelial cells, all the groups showed reduced positive staining in the defect area compared to the WT control group (Figure 3A). Further, DB mouse calvarial regeneration is associated with reduced vascularization that was not overcome by the addition of WT macrophages in the collagen scaffolds.

To evaluate if the effect of macrophages was mediated by paracrine effects of the macrophage secretome, we performed an *in vitro* mesenchymal stem cell (MSC) differentiation assay in the presence of WT and DB macrophage-conditioned medium (wtCM and dbCM, respectively) and assessed ALP activity. Results presented in Figure 3C indicate that ALP activity increased when the MSCs were subjected to an osteogenic differentiation medium with respect to a growth medium. This increase remained unchanged in the presence of wtCM and was significantly reduced (by ~30%) in the presence of dbCM, indicating the negative effects of the DB macrophage secretome on MSC differentiation.

**Figure 4:**
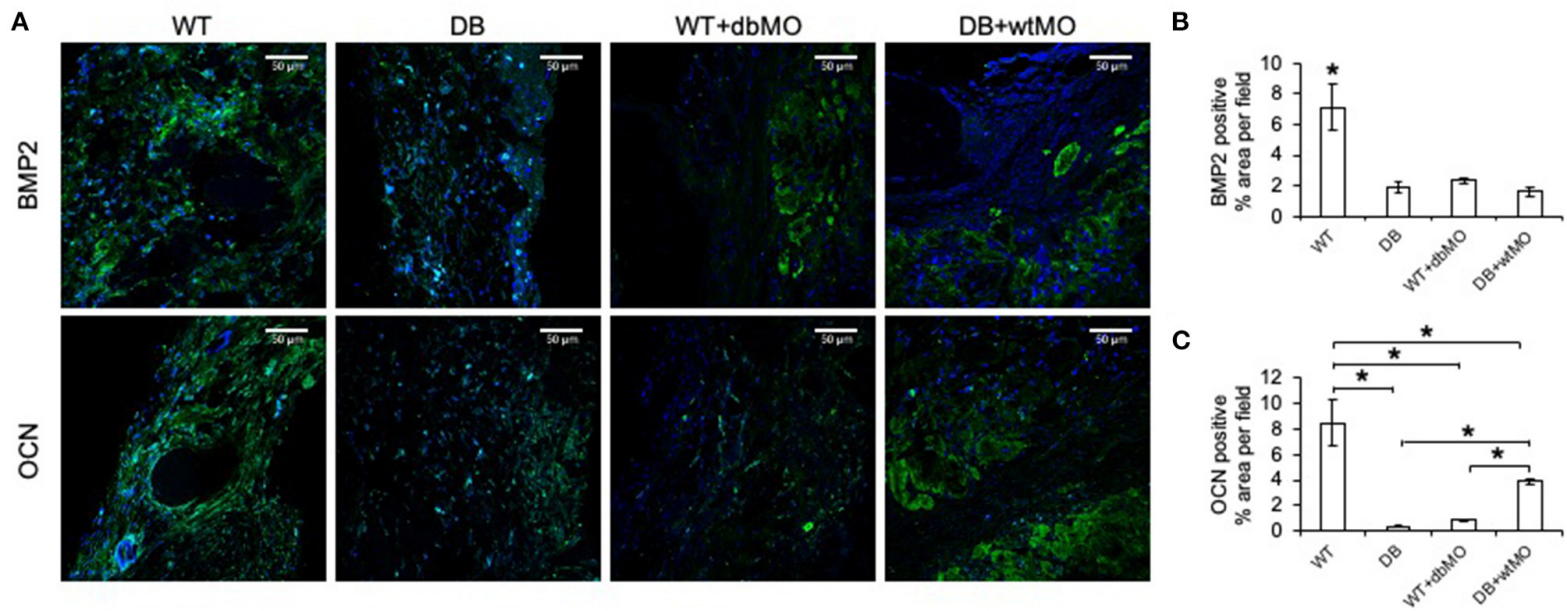
Fluorescent IHC of calvarial sections for BMP2 and OCN. **(A)** Representative confocal micrographs of calvarial sections immunostained for BMP2 (green) and OCN (green). Note the generalized expression of BMP2 and OCN in WT and the relative absence of either protein in DB or WT mouse with the DB macrophages (WT+dbMO) group. Scale bar represents 50 μm in all images. **(B,C)** Quantitation of BMP2- and OCN-positive areas as percentage per field view (*n* = 4). *Statistical significance (*P* < 0.05) as measured by Tukey's *ad hoc* test post ANOVA. WT, wild-type mice; DB, db/db mice; WT+dbMO, WT mice treated with DB macrophages; DB+wtMO, DB mice treated with WT macrophages.

Further proof of macrophage-associated control of bone regeneration was obtained by IHC evaluation of two key osteogenic protein expressions, namely, BMP2, and OCN (Figure 4A). Comparative IHC staining of these proteins in wounds from the four groups of mice demonstrated the relatively low abundance of either protein in the DB mouse defects and WT defects treated with collagen containing DB macrophages (Figures 4B,C). This finding is aligned with the relative absence of incipient bone formation observed in the groups of DB mouse and DB mouse treated with WT macrophages. While BMP2 expression was not significantly improved by treatment of DB mouse defects with WT macrophages, OCN expression levels were significantly improved compared to DB group and the WT group treated with DB macrophages, indicating partial rescue of OCN expression.

**Figure 5:**
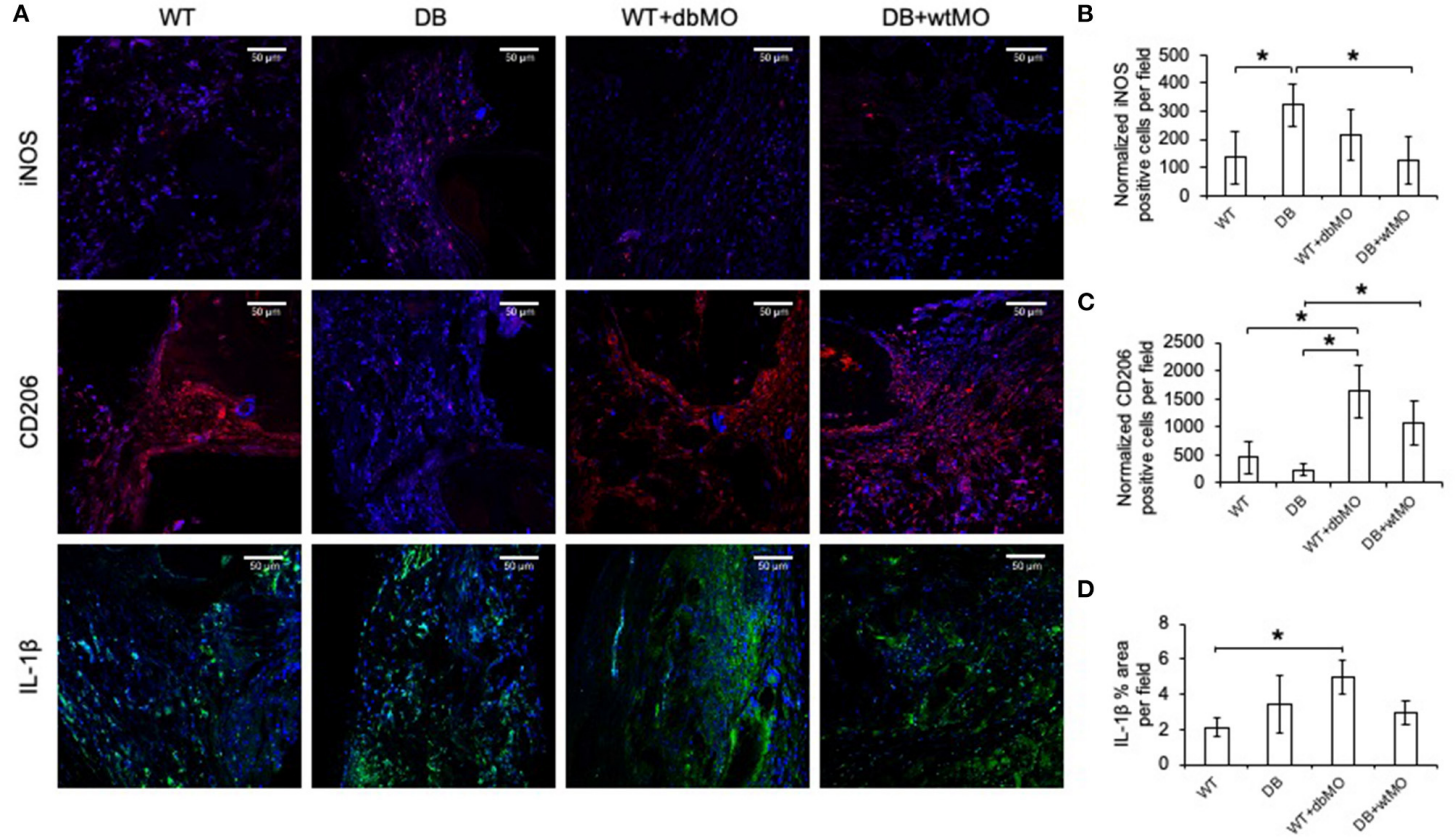
Fluorescent IHC of calvarial sections for iNOS, CD206 and IL-1β. **(A)** Representative confocal micrographs of 4-week calvarial sections stained for iNOS (red), CD206 (red), and IL-1β (green). **(B,C)** Quantitation of iNOS- and CD206-positive cells as percentage per field view (*n* = 4). Note the increase of iNOS-positive cells and the absence of CD206-positive cells in the DB group and the increase in the number of CD206-positive cells in the DB with WT macrophages (DB+wtMO) group compared to the DB group. **(D)** Quantitation of the IL-1β-positive area as percentage per field view (*n* = 4). In all images, nuclei are stained with DAPI. Scale bar represents 50 μm in all images. *Statistical significance (*P* < 0.05) calculated by Tukey's *ad hoc* test post ANOVA. WT, wild-type mice; DB, db/db mice; WT+dbMO, WT mice treated with DB macrophages; DB+wtMO, DB mice treated with WT macrophages.

Given the relative inflammatory nature of the isolated DB macrophages (Figures 1, 2), we sought to define the inflammatory status of macrophages in healing calvarial defects. Figure 5A illustrates the confocal microscopic evaluation of iNOS, IL-1β-positive (M1-like), and CD206 (M2-like) macrophages in healing the calvariae in the four treatment groups. The quantitation of expression of these proteins is represented in Figures 5B–D. The iNOS/CD206 (M1/M2) ratio was 4.56-fold in the DB group, whereas it was 0.45-fold in WT group treated with DB macrophages and 0.4-fold in DB group treated with WT macrophages with respect to the WT group. iNOS and IL-1β were present in significantly increased amounts in the DB group compared to the WT group. When WT macrophages were introduced into the DB mouse wounds, the expression levels of both iNOS and IL-1β were significantly reduced, suggesting a reduction in inflammation in these groups. Conversely, when DB macrophages were added to WT defects, the expression levels of iNOS and IL-1β were increased. While the iNOS expression increase was not statistically significant, a significant increase in IL-1β expression was observed. With respect to CD206, a marker for M2-like cells, DB mice displayed a reduced presence of this marker, and its expression increased with the addition of WT macrophages to DB wounds. Interestingly, the addition of DB macrophages to WT wounds dramatically increased the expression levels of this marker. Relatedly, the relative absence of CD206 staining in DB mouse calvarial defects suggests a reduced level of reparative macrophages that is associated with the absence of incipient bone repair in this group However, the treatment of either DB mouse calvarial defects with WT macrophages or WT defects with DB macrophages was associated with an increased number of CD206-positive cells at the defect sites of both types of animals. The introduction of WT macrophages to the diabetic wounds shifted this ratio toward normalcy and was associated with improved incipient bone repair. The diabetic bone defects showed impaired early healing that was associated with the presence of the inflammatory state of the macrophages and the altered ratio of inflammatory to reparative macrophages.

### Possible Role of miRNA in the Function of WT and DB Macrophages

**Figure 6:**
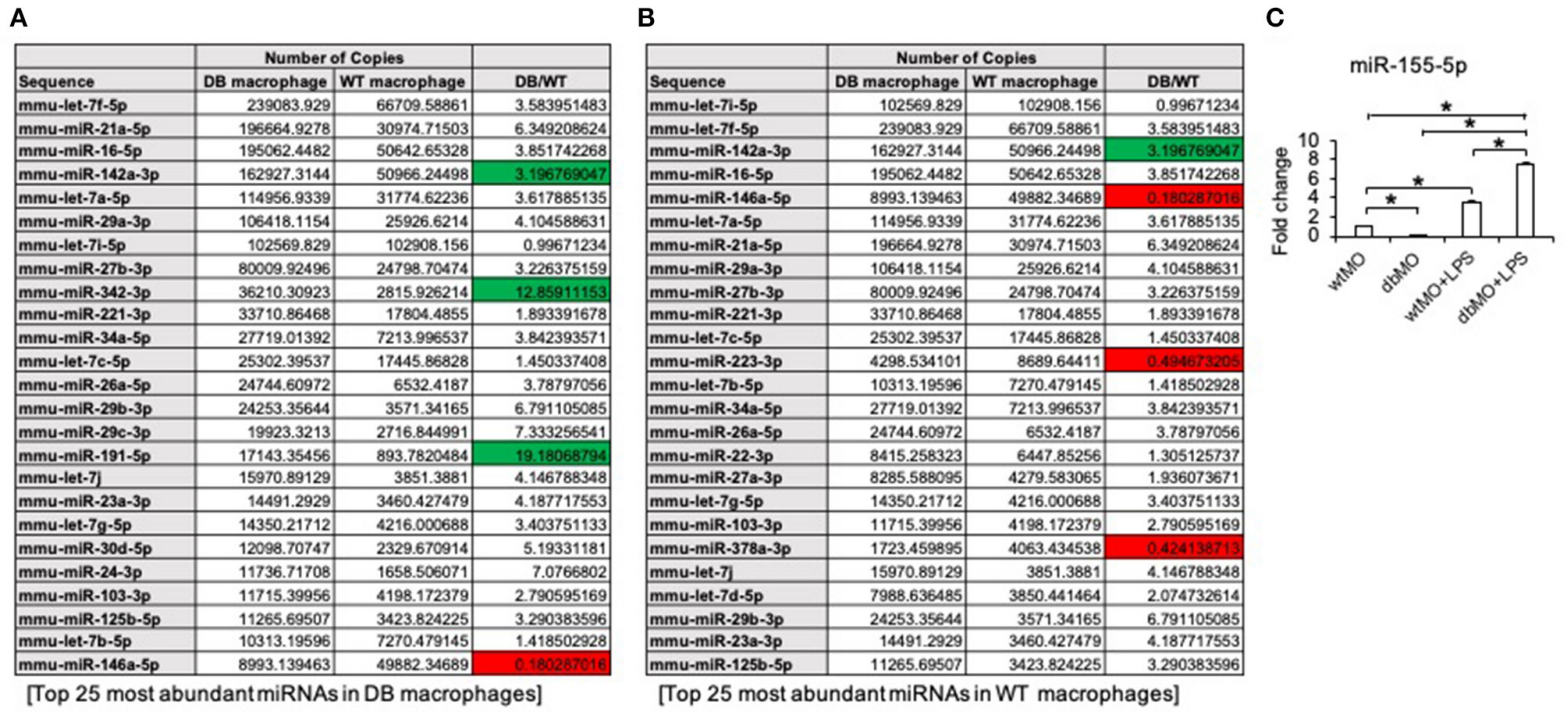
miR-Seq analysis of WT and DB macrophages. **(A)** A list of the top 25 most abundant miRNAs in DB macrophages and their expression levels in WT macrophages. **(B)** Top 25 most abundant miRNAs in WT macrophages and their expression levels in DB macrophages. The pro-inflammatory miRNAs are highlighted in green, and anti-inflammatory miRNAs are highlighted in red. Note that the increased expression of pro-inflammatory miRNAs (miR-142a-3p, miR-342-3p, and miR-191-5p) and suppressed expression of anti-inflammatory miRNAs (miR-146a-5p, miR-223-3p, and miR-378a-3p) in DB macrophages compared to WT. **(C)** Quantitative RT PCR of WT macrophages (wtMO) and DB macrophages (dbMO) for miR-155-5p. The graph showing the expression level of miR-155-5p in WT and DB macrophages with or without LPS stimulation. Data represent mean fold change with respect to WT macrophage expression ± SD (*n* = 4). Note the significant increase of miR-155-5p expression upon LPS treatment in DB macrophages (dbMO+LPS). *Statistical significance (*P* < 0.05) calculated by Tukey's *ad hoc* test post ANOVA.

Our results indicated that the resting states of the WT and DB macrophages are varied and that their response to pro-inflammatory stimulus is different. To evaluate the possible role of miRNAs in WT and DB macrophages, we performed miR-Seq analysis of the two isolated cell types. The table presented in Figure 6A lists the top 25 most abundant miRNAs in DB macrophages and their expression levels in WT macrophages. Figure 6B lists the top 25 miRNAs in WT macrophages and their corresponding expression in DB macrophages. The green color coding denotes pro-inflammatory miRNA showing increased expression in DB macrophages compared to WT, and the red color coding denotes anti-inflammatory miRNA showing reduced expression in DB macrophages compared to WT. In addition to these miRNAs, we also evaluated the expression of miR-155-5p in WT and DB macrophages by qRT PCR and its change in the presence of LPS treatment. miR-155 is characterized as a master regulator of inflammation (Mahesh and Biswas, 2019), and we have demonstrated the role of exosomal miR-155 in bone repair. miR-155-5p was downregulated in resting DB macrophages (Figure 6C). However, upon LPS stimulation, DB macrophages showed a more robust increase in miR-155-5p compared to WT macrophages, indicating their propensity for an enhanced inflammatory response to stimuli.

## Discussion

T2DM is a disease of epidemic proportion affecting ~10.5% of the US population (34.2 million people in 2020) (Centers for Disease Control and Prevention, 2020). It is characterized by impaired insulin secretion, glucose intolerance, and hyperglycemia (American Diabetes Association, 2010). As a result, multiple organ systems are affected and cause significant comorbidities. A common underlying sequela of T2DM is immune dysregulation and inflammation. In addition to changes in the T-cell compartment and reduction in regulatory T cells and NK cells, there is reported abnormal polarization of macrophages (Zhou et al., 2018). The alterations in immune cell function in T2DM may have subsequent effects on other tissues and generally manifest as challenges in wound healing (Barman and Koh, 2020). The DB macrophage displays a pro-inflammatory phenotype as shown above and suggests that this diabetes-related macrophage phenotype is involved in the modulation of bone repair. This may explain, in part, how engraftment of the DB macrophage impaired bone regeneration in WT mice.

This finding of reduced bone regeneration in DB mice and the finding that DB macrophages impair bone regeneration in WT mice are consistent with the existing evidence that bone healing is impaired in T2DM. A 2015 review indicated the increased risk of fracture in T2DM and suggests that the impairment of bone formation is associated with increased osteoblast apoptosis and reduced expression of osteoinductive factors (Marin et al., 2018). A more recent review has also highlighted the effect of T2DM on MSC differentiation leading to alterations in vascularization and increased number of adipocytes, which negatively impact bone regeneration (Cassidy et al., 2020).

The number of bone marrow MSCs is reduced in T2DM (Cassidy et al., 2020), suggesting a systemic role for heightened inflammation in bone regeneration. A differential gene expression study of blood from T2DM and non-diabetic patients revealed low bone morphogenetic protein 4 (*BMP4*), *BMP7*, and *RUNX2* expression in T2DM patients. Further, metformin was observed to enhance *BMP4* levels and osteogenic function of MSCs (Liang et al., 2020). Impaired bone repair in diabetics, demonstrated in this model of bone repair in T2DM, may be influenced by reduced numbers or activity of MSCs in osteogenesis.

The db/db mouse model is a recognized model of T2DM. Previous studies of bone regeneration conducted in this model indicate that db/db mice display reduced bone repair (Wallner et al., 2015). For example, using a 1-mm unicortical defect, absence of bone regeneration was recovered by treating defects with syngeneic adipogenic stem cells (Wallner et al., 2016). While suggesting that bone regeneration was recovered by augmenting the number of osteoinductive stem cells, the tibia defects are not critical-sized defects, and potential paracrine effects of the implanted cells were not considered. Obesity does complicate the interpretation of studies with db/db mice. Obesity-related inflammation, however, does increase the pro-inflammatory phenotype of macrophages. While obesity is a comorbidity of diabetes, we did not intend for this work to explain obesity-mediated effects on macrophage function in bone repair. To separate the effects of obesity from T2DM, further studies need to be performed wherein the effects observed in db/db mice are directly compared to those in non-diabetic obese mice. In this study, we isolated macrophages from DB mice and placed them in WT wounds. These WT animals were not obese and yet displayed altered healing when engrafted with DB macrophages. Based on these observations, we demonstrated that the macrophage of the DB mouse altered bone repair associated with their altered inflammatory status (M1/M2 ratios).

T2DM-related impairment of osteogenesis has been previously observed in the Zucker diabetic fatty (ZDF) rat calvarial model in which partial bone regeneration was observed at 8 weeks, but significantly reduced amounts of bone with reduced angiogenesis were found in critical-sized defects of the diabetic rat calvaria (Caliaperoumal et al., 2018). Separate studies in the ZDF rat indicate that osteoblastogenesis is suppressed in T2DM (Hamann et al., 2011). Not only are osteoblasts affected, osteoclastogenesis and osteoclast activity is also reduced in a rat model of T2DM (Hu et al., 2019). The early time point investigated in this study did not warrant a study of osteoclastogenesis. However, this serves as a limitation of this study, and further studies are required to ascertain the role of diabetic macrophages in osteoclastogenesis and bone resorption.

The macrophage is a known significant factor in T2DM pathophysiology. The monocyte/macrophage population contributes to chronic inflammation that impairs wound healing and may reflect a greater number of macrophages and their dysregulation (Barman and Koh, 2020). Studies that deplete monocytes/macrophages from mice (clodronate treatment or MaFIA mice) demonstrate that the absence of macrophages impairs bone regeneration (Davison et al., 2014; Kaur et al., 2017). Based on the concept that macrophages are one of the primary regulators of wound healing, the present investigation involved the syngeneic transplantation of DB macrophages in WT wound that resulted in impaired osteogenesis. The larger number of macrophages and their relatively pro-inflammatory phenotype contributed to the reduction in bone repair in the WT mice when DB macrophages were implanted in them. Conversely, transplantation of WT macrophages into the DB mouse partially reconstituted the impaired regenerative process in these diabetic osseous wounds. We speculate that this partial restoration may be attributed to the reduction in the inflammatory status of the wound bed, resulting in altered M1/M2 ratios with an increase in the M2 macrophage population favoring bone repair.

While the results of this study are solely focused on the influence of diabetic macrophages (as indicated here by increased numbers of CD206-positive cells) in bone repair, as discussed previously, other effects of T2DM should also be considered. For example, the role of insulin resistance requires further investigation. A recent study identified that insulin resistance as a result of T2DM negatively affects bone regeneration (Srikanthan et al., 2014). On the other hand, the anabolic role of insulin in bone formation has also been documented and reviewed (Thrailkill et al., 2005). This dichotomy is currently debated at molecular and mechanistic levels, and it would be interesting to investigate the role of macrophages in this process. We envision that the results presented here outlining the role of diabetic macrophages may serve as a starting point for such investigations.

In this large, critical-sized defect, islands of osteogenesis formed remotely from the bone surfaces populated by bone-specific macrophages termed osteomacs (Miron and Bosshardt, 2016; Batoon et al., 2017) are likely less influenced by these regulatory macrophages that form canopy structures above progenitor cells lining the bone surface. Circulating cells also contribute to wound healing, and studies in the parabiotic mouse model demonstrate the contribution of circulating cells to osteogenesis in fracture repair (Kumagai et al., 2008), indicating that large defects are populated by cells derived from the circulation. Here, as in other wounds, macrophages are likely derived from circulating monocytes (Rodero et al., 2014).

The implanted macrophages may exert their effects on osteoprogenitors in the local environment by both direct cell–cell and soluble factor signaling (Champagne et al., 2002; Pajarinen et al., 2019). ALP activity was reduced in differentiating MSCs in the presence of dbCM when compared to culture in the presence of wtCM. The measured differences in BMP2 and OCN expression further imply that the observed absence of incipient bone formation in the DB mice or in WT mice treated with DB macrophages is due, in part, to paracrine effects of the DB macrophage upon the osteoprogenitor cells. Factors secreted by macrophages and known to reduce OSTERIX and BMP2 expression include IL-1β, IL-6, and TNFα (Nakase et al., 1997). Of note, all these pro-inflammatory factors' gene expression was elevated in the DB macrophages relative to the WT macrophages, again suggesting that the macrophage phenotype of the DB mouse directly impairs osteoblastogenesis.

In addition to growth factors, cytokines, and chemokines, cells secrete exosomes [30- to 150-nm extracellular vesicles containing protein and miRNA cargo (Golchin et al., 2018)] that transfer their cargo as regulatory signals from parental to target cells. Exosomes serve as a carrier for miRNA from parental cells to target cells, and exosomal miRNAs are implicated as the primary effectors of exosome function (Huang et al., 2020). In a recent study, we have highlighted the role of macrophage-derived exosomal miRNA in bone repair (Kang et al., 2020). In this manuscript, we have performed a miR-Seq analysis of the miRNA composition of macrophages from WT and DB mice. Of the top 25 most abundantly expressed miRNAs in the WT and DB macrophages, we have identified miRNA candidates that have proven roles in inflammation. Notably, our results indicate the upregulation of pro-inflammatory miRNAs (Wei et al., 2013; Gu et al., 2017; Mandolesi et al., 2017) and the downregulation of anti-inflammatory miRNAs in DB macrophages (Rückerl et al., 2012; Testa et al., 2017; Cheng et al., 2020; Kang et al., 2020), indicating that the transfer of miRNA via paracrine mechanisms may also play a role in macrophage-mediated control of bone repair. In addition to the miRNA presented in the table, we have also evaluated the changes in expression levels of miR-155. miR-155 is considered a master regulator of inflammation affecting the NLRP3 inflammasome pathway (Mahesh and Biswas, 2019). The stimulated macrophage expression of miR-155 in DB macrophages is greater when compared to WT macrophages, further indicating the sensitized pro-inflammatory state of the macrophage phenotype in DB mice. Further studies on the role of diabetic macrophage miRNAs and the paracrine role of these miRNAs via exosomes in relation to bone repair and in general tissue repair are warranted to understand this complex process in greater detail.

## Conclusion

The role of macrophages in the pathology of bone healing observed in diabetes was modeled in the DB mouse. Transfer of DB macrophages to WT mouse calvarial defects impaired incipient bone repair *in vivo* and affected M1/M2 ratios in the wound bed, and the macrophage-derived conditioned medium impaired ALP activity in differentiating MSCs *in vitro*, suggesting that the paracrine effects of macrophages on incipient osteogenesis may impair bone formation in the DB mouse. The partial restoration of incipient bone formation in the DB mouse by transfer of WT mouse macrophages to calvarial wounds further suggests that macrophages play an important role in the impairment of osteogenesis in diabetes. Targeting the inflammatory phenotype of the diabetic macrophage may provide an alternative therapeutic strategy to enhance bone repair in diabetic patients.

## Data Availability Statement

The datasets presented in this study can be found in online repositories. The names of the repository/repositories and accession number(s) can be found at: BioProject ID PRJNA685165.

## Ethics Statement

The animal study was reviewed and approved by Animal Care Committee of the Office of Animal Care and Institutional Biosafety (OACIB) of the University of Illinois at Chicago (protocol number 20-020).

## Author Contributions

LC and MK conceived of the project and experimental design. MK, GT, C-CH, YL and SS contributed to the laboratory and animal experiments. LC, SR, and MK prepared this manuscript. All authors contributed to the article and approved the submitted version.

## Conflict of Interest

The authors declare that the research was conducted in the absence of any commercial or financial relationships that could be construed as a potential conflict of interest.
